# Cross-cultural adaptation and psychometric properties of the short version of COPSOQ II-Brazil

**DOI:** 10.11606/s1518-8787.2021055003123

**Published:** 2021-10-13

**Authors:** Josiane Sotrate Gonçalves, Cristiane Shinohara Moriguchi, Thaís Cristina Chaves, Tatiana de Oliveira Sato

**Affiliations:** I Universidade Federal de São Carlos Centro de Ciências Biológicas e da Saúde Departamento de Fisioterapia São CarlosSP Brasil Universidade Federal de São Carlos. Centro de Ciências Biológicas e da Saúde. Departamento de Fisioterapia. São Carlos, SP, Brasil

**Keywords:** Job Satisfaction, Occupational Health, Psychological Tests, Surveys and Questionnaires, Translating, Validation Study

## Abstract

**OBJECTIVES:**

Translate and culturally adapt the short version of Copenhagen Psychosocial Questionnaire II (COPSOQ II) into Brazilian Portuguese (COPSOQ II-Br) and evaluate its psychometric properties.

**METHODS:**

Translation and cultural adaptation followed the standardized guidelines. Structural validity was assessed using exploratory factorial analysis. Test-retest reliability was evaluated by intraclass correlation coefficient (ICC_3,1_) and internal consistency by Cronbach’s alpha. Floor and ceiling effect was considered acceptable if less than 15% of participants reported the lowest or highest scores. Measurement error was assessed by standard error of measurement (SEM), while construct validity was tested by correlating the COPSOQ II-Br, the Job Content Questionnaire and the Nordic Musculoskeletal Questionnaire.

**RESULTS:**

The study evaluated a total of 211 civil servants and service providers in the test and 157 in the retest. After cross-cultural adaptation, the COPSOQ II-Br structure comprised seven domains and 11 dimensions. Most dimensions showed acceptable floor and ceiling effects, excepting “Work family conflicts” (floor effect of 26.1%), and “Meaning and commitment” and “Job satisfaction,” with ceiling floor of 27.5% and 22.3%, respectively. Cronbach’s alpha values reached the recommended levels (varied between 0.70 and 0.87). Test-retest reliability indicated that all dimensions had ICC between 0.71 and 0.81. SEM ranged from 0.6 to 2.2 and the construct validity showed good results with the tested instruments (significant positive and negative correlations).

**CONCLUSIONS:**

All psychometric properties of the short version COPSOQ II-Br are suitable for use in Brazil. The instrument is thus validated and can be used by occupational health and human resources professionals to evaluate psychosocial working conditions.

## INTRODUCTION

Research on mental disorders has increased worldwide given its growing impact on workers’ health, wellbeing and productivity^1,2^. Between 2006 and 2017, Brazil registered 8.474 cases of work-related mental disorders, with severe stress reactions and adjustment disorders being the most common diagnoses (47%), followed by depressive episodes (24%) and other anxiety disorders (17%)^3^.

Mental disorders can be caused by psychosocial risk factors such as high work and emotional demands, low decision authority, low social support, high work efforts and obligations, low rewards (remuneration, recognition and career opportunities), and feelings of injustice^4^. Exposure to these factors can compromise healthy functioning at the organic, emotional, cognitive, social, and behavioral levels^5^.

Psychosocial risk factors can be assessed using questionnaires, a widely employed practice since its application and data analysis is relatively easy and cheap^6,7^.

One such questionnaire is the Copenhagen Psychosocial Questionnaire (COPSOQ), developed in Denmark and translated, adapted, and tested in several countries^8^. Based on a multifaceted approach, unlike other questionnaires^15,16^, the instrument covers seven psychosocial theories: the Job Characteristics Model, the Michigan Organization Stress Model, the Job Demands-Control Model, the Sociotechnical Approach, the Action-Theoretical Approach, the Effort-Reward Imbalance Model, and the Vitamin Model^15^. Made available in 2005^15^, COPSOQ I was then revised in 2010 (COPSOQ II) to include other relevant^16^ and less studied dimensions. In 2019, the International Network made the COPSOQ III available (https://www.copsoq-network.org/) to facilitate the communication and international use of the instrument^17^.

Created to address important work-related aspects such as recognition, justice, and trust and to incorporate aspects brought forth by the experience of applying COPSOQ I, COPSOQ II also had a short version designed to be applied in the workplace^16^. In Brazil, the medium version of COPSOQ I was translated and validated^18^, as was the Spanish version of COPOSOQ II – COPSOQ-ISTAS21 II^19^.

However, we still lack studies on the cross-cultural adaptation of the short version of COPSOQ II into Brazilian Portuguese. In this sense, due to its comprehensive characteristics and great utility for organizations, making a Brazilian Portuguese version of this instrument available to occupational health professionals is paramount.

This study proposed, thus, to translate and cross-culturally adapt the short version of COPSOQ II and to evaluate the psychometric properties of the Brazilian Portuguese version (COPSOQ II-Br) – structural validity, test-retest reliability, internal consistency, floor and ceiling effect, measurement error and construct validity.

## METHODS

### Study Design

This cross-sectional study was conducted according to the recommendations of the COnsensus-based Standards for the selection of health Measurement Instruments (COSMIN) for analysis of psychometric properties^20^ and according to the stages of cross-cultural adaptation^21^. The research project was approved by the Human Research Ethics Committee (CAAE: 64255917.7.0000.5504; Opinion number: 1.912.156).

### Participants

The study took place in the city of São Carlos in several productive sectors from October 2016 to September 2018, using a non probabilistic sample of civil servants and service providers in the areas of education, health, administration and human resources, general services (cleaners and conservation), maintenance and repair (building assistance and zookeepers). The inclusion criteria were: workers between 18 and 65 years of age^14,16^, with at least six months on the job and a work routine of at least 20 hours a week (4 hours/day, 5 days a week). All participants were informed about the study and those who agreed to participate signed an Informed Consent Form (ICF).

**Figure 1:**
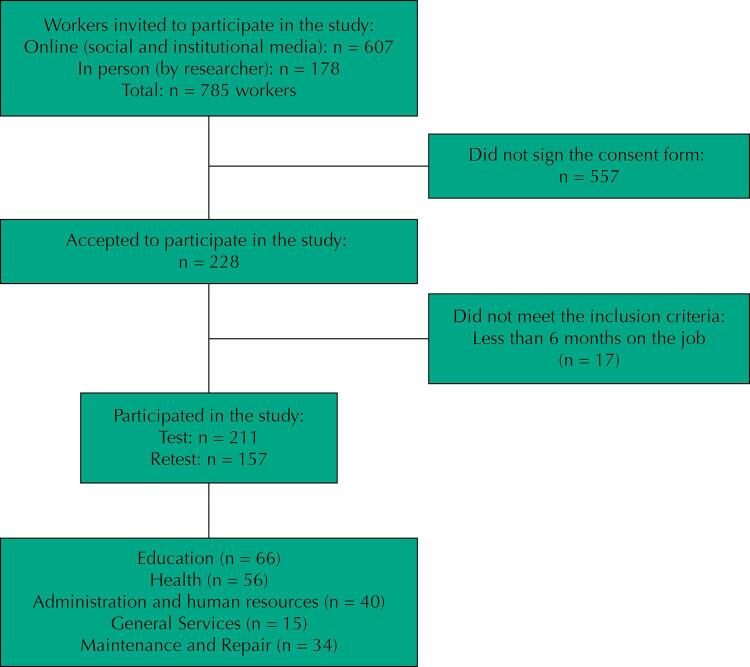
Study flowchart.

A total of 785 workers were invited to participate in the study. Of the 228 who agreed to participate, 211 were included in the study (Figure 1).

### Instruments

#### COPSOQ II

COPSOQ comprises seven conceptual models to identify psychosocial risk factors, and all versions have dimensions that measure exposure indicators (risks), health effects, satisfaction and stress^5,15,16^.

COPSOQ II has three versions: long (41 dimensions and 127 questions), medium (28 dimensions and 87 questions) and short (23 dimensions and 40 questions)^16^. In the short version, questions are answered according to a 5-level Likert scale, with question 1B being the only one with an inverted value. Score is calculated by adding up the responses of each dimension, except for the domain offensive behaviour^16^. According to the number of questions in each dimension, the score can vary from 0 to 3, 0 to 4, 0 to 6 or 0 to 8 points. For each dimension, the values obtained can be classified as favorable (green), require attention (yellow) and unfavorable (red)^5^. Another classification method involves calculating the average value of each dimension.

#### Job Content Questionnaire (JCQ)

JCQ is a standardized instrument proposed to measure the dimensions of the demand-control-social support model^22^. The short and adapted version into Brazilian Portuguese consists of 17 questions covering psychological demand, control, and social support, evaluated by a Likert scale. Score is obtained by adding the questions that make up the dimension, with demand varying between 5 and 20 points, control between 6 and 24 points, and social support between 6 and 24 points^22^.

#### Nordic Musculoskeletal Questionnaire (NMQ)

NMQ is used to identify the presence of musculoskeletal symptoms in the past seven days and 12 months, seeking health care and having functional limitations due to these symptoms^23^. Participants report the presence of musculoskeletal symptoms such as pain, numbness or discomfort in the neck, lower back, shoulders and other areas in the past 12 months and seven days, on a dichotomous scale (presence or absence)^23^.

## Cross-Cultural Adaptation and Psychometric Properties of COPSOQ II-Br

### Cross-cultural adaptation of COPSOQ II-Br

After obtaining the approval for translation, the process of translation and cross-cultural adaptation followed 5 stages^21^:

1. Translation from English into Brazilian Portuguese (a layman translator and an expert translator, both bilingual and Brazilian Portuguese native speaker, worked independently);
2. Synthesis of translation (synthesis of the translations made by two experts in the field of ergonomics, discussions of differences and consensus among translators);
3. Back-translation (made by two English native speaker specialist translators without prior access to COPSOQ content);
4. Meeting with a committee of specialists for the pre-final version (professionals trained in ergonomics, language professionals, researchers specialized in cross-cultural adaptation and translators);
5. Pre-test (2 pre-tests, where participants were interviewed and the understanding of the questionnaire items assessed).

The final version of the COPSOQ II-Br short version was thus developed and cross-culturally adapted into Brazilian Portuguese. After this 5-step process, we finally tested the psychometric properties of the instrument.

## Procedures

Data collection included in-person and online (invitation via link on social and institutional media) questionnaires^14^. In-person data collection took place both in the process of cross-culturally adaptating the instrument into Brazilian Portuguese (pre-test) and in the evaluation of psychometric properties. Online data collection was conducted only for the measurement properties. We chose one or the other according to the participants’ schooling level, e.g., cleaners, building assistance and zookeepers were unfamiliar with answering online forms. First, the participants received the ICF and those who consented were included in the study. Then, after a explanation about the questions and guarantee of confidentiality, a researcher sent the questionnaires to the participants, or devilered them in person. The questionnaire is self applicable. Doubts about the questionnaire were solved in the workplace and via e-mail (online format).

To assess test-retest reliability, we perfomed two data collection with an interval of two weeks between measurements to ensure that no changes had occurred^24^. Participants received e-mails reminding them of their participation in the study, with a five-day deadline set for the retest response.

To assess construct validity, we applied the NMQ in the testing phase and the JCQ in the retest, so that the evaluations would not become extensive. The participants completed the questionnaires without additional hours of work or sallary reduction.

### Psychometric properties of COPSOQ II-Br

#### Structural validity

Structural validity concerns the degree to which the scores of an instrument adequately reflect the dimensionality of the construct to be measured^20^. Exploratory factor analysis determines whether items compose one or more dimensions and should be carried out when internal consistency is adequate or in the impossibility of conducting confirmatory analysis^24^. The present study carried out a exploratory factor analysis, considering the same sample as that used to assess internal consistency, i. e., a minimum of 100 participants^20,24^.

Principal Component Analysis (PCA) with a final solution based on the Varimax orthogonal rotation was used to evaluate the internal structure of the measurement. The relevance of performing a factor analysis was evaluated by the sample adequacy ratio *Kaiser-Meyer-Olkin* (KMO)^13,25^. Retained factors were derived according to the magnitude of the *eigenvalues* (greater than 1) and the proportion of extracted variance. Scree plot was used to determine the number of factors to be extracted. When items had low loads (less than 0.4) in more than one component (complex structure), they were removed from the analysis^25^. A forced analysis with one or two factors was performed when results indicated more than two factors^13,25^.

#### Internal consistency

Internal consistency assess the degree of interrelation between items, i. e., whether the items are measuring the same concept^20^. We considered a minimum sample of 100 participants to ensure the stability of the variance-covariance matrix and evaluate the internal consistency^24^. Cronbach’s alpha was calculated separately for each dimension using baseline data (n = 211). Scores between 0.70–0.95 indicate acceptable internal consistency^24^.

#### Floor and Ceiling Effect

Floor and ceiling effects reflect problems with content validity and can limit detecting changes over time^24^. These were considered acceptable if less than 15% of participants reported the lowest or highest scores^24^. Distribution of the dimensions is presented in mean, standard deviation (SD), median and interquartile range (IQR).

#### Test-retest reliability

Reliability is the degree to which a measurement is free of measurement error, and if the scores are identical in repeated measurements over time (test-retest) for the same participants^20^. Studies recommend a minimum sample of 50 participants to test the reliability measure^24^. Test-retest reliability was assessed using the intraclass correlation coefficient (ICC_(3,1)_), with ICC values greater than 0.70^24^indicating reliability.

#### Measurement error

Measurement error means the systematic and random error of a participant’s score that is not assigned to real changes in the construct being measured^20^, which requires a minimum sample of 50 participants^24^. This psychometric property was estimated by calculating the standard error of measurement (SEM) at baseline, using the following formula: SEM = SD*√(Cronbach’s alpha-1), where SD is the standard deviation of all the participants’ baseline scores^26^. Minimal detectable change (MDC) with 90% confidence was calculated by the following formula: MDC = 1,64*SD*√ (2 (1-R)), where R is the test-retest ICC value and SD the standard deviation of all the participants’ baseline scores. A change equal to or greater than the MDC, before and after an intervention, indicates that there has been a real change at the individual level^26^.

#### Construct validity

Construct validity reflects the degree to which an instrument score is consistent with hypotheses, based on the assumption that the instrument measures the construct under assessment^20^. This psychometric property was tested between COPSOQ II-Br and JCQ, which also assesses psychosocial aspects, and NMQ, as musculoskeletal symptoms are closely related to psychosocial aspects. Musculoskeletal pain is common in the working population and has been associated with high work demands and low control^27^.

Spearman’s correlation coefficient (r_s_) was calculated to assess the construct validity between the JCQ scales^22^ and dimensions of the COPSOQ II-Br. The relation between symptoms in the neck and lower back in the past 12 months and seven days using the NMQ^23^ and the dimensions of COPSOQ II-Br was assessed by point-biserial correlation coefficient (r_pb_). We also tested the Spearman’s (r_s_) correlation between the number of areas with musculoskeletal symptoms in the past 12 months and seven days and the dimensions of COPSOQ II-Br. Correlation coefficients greater than 0.70 were considered strong, between 0.70 and 0.50 moderate, and less than 0.50 weak^24^.

Regarding the expected relationship between the tested instruments, we formulated the following hypotheses: 1) there will be a significant and positive correlation between the dimension “Demands at work” of COPSOQ II-Br and the “Demand” scale of JCQ; 2) there will be a significant and positive correlation between the dimensions “Influence and development” and “Meaning and commitment” of COPSOQ II-Br and the “Control” scale of JCQ; 3) there will be a significant and positive correlation between the dimensions “Interpersonal relationships” and “Leadership” of COPSOQ II-Br and the “Social support” scale of JCQ; 4) there will be a significant and positive correlation between the dimensions “Demands at work”, “Work family conflict” and “Burnout and stress” of COPSOQ II-Br and musculoskeletal symptoms; 5) there will be a significant and negative correlation between the dimensions “Interpersonal relationships”, “Job satisfaction” and “General health” of COPSOQ II-Br and musculoskeletal symptoms.

The data were analyzed using SPSS software version 22.0 (SPSS Inc, Chicago, IL, EUA).

## RESULTS

### Sample Characteristics

Our study sample comprised mostly women (71%), with mean age of 40 years (SD: 11), married/living with a partner, with more than 15 years of study and up to 2 years working in the same company. Table 1 presents the complete sociodemographic characteristics of the sample.

**Table 1:** Characteristics of the participants (n = 211).

Variables	n	%
Sex		
Female	150	71.1
Male	61	28.9
Age		
18 to 20 years	2	1.0
21 to 30 years	27	12.8
31 to 40 years	83	39.3
More than 40 years	99	46.9
Marital status		
Single, divorced or widowed	89	42.2
Married or living with partner	122	57.8
Schooling level		
9 to 10 years of study	29	13.7
10 to 12 years of study	51	24.2
13 to 15 years of study	40	19.0
> 15 years of study	91	43.1
Time in institution/company^a^		
6 to 11 months	14	6.6
1 to 2 years	103	48.8
2 years to 5 years	26	12.3
> 5 years	65	30.8
Hours of work/day		
4 to 6 hours/day	4	1.9
7 to 9 hours/day	184	87.2
> 10 hours/day	23	10.9
Work shift		
Morning	183	86.7
Night	28	13.3

^a^ missing data (n = 3).

### Cross-Cultural Adaptation Process

We kept the format of the original questionnaire, with minor changes. Discrepancies were resolved by consensus between translators and by the expert committee.

We conducted two pre-tests. In the first, a sample of 30 workers from education (50%) and cleaning services (50%), with high and low level of education, respectively, answered the instrument. Workers (57% of the sample) had difficulty in answering question 4A due to semantics. Since the percentage of doubt exceeded 20%^27^, the requestion was reprhased (from “Do you have a high degree of influence in relation to your work?” to “Do you have a high degree of influence in decisions about your work?”). A sample of 30 workers from the education, cleaning and maintenance/repair services, 15 women (50%) and 15 men (50%) with high and low level of education (50% in each group), participated in the second pre-test. At this stage, workers encountered difficulties only with questions 1A, 4A, 10A. As only 7% of workers had doubts on each question, no redesign was necessary^27^. Based on the results of the pre-test, we finally proposed a short version of COPSOQ II culturally adapted to Brazilian Portuguese (COPSOQ II-Br)^a^.

### Psychometric Properties

#### Structural validity

PCA identified 11 factors, which represent approximately 50% of the variance (Table 2). The “Demands at work” domain comprised 6 questions and the factorial solution contained a factor (KMO = 0.72). “Work Organization and content” extracted two factors (KM = 0.70), the first being “Influence and development” and the second, “Meaning and commitment”. The “Interpersonal relationships and leadership” domain also obtained a solution with two factors (KMO = 0.87) (“Interpersonal relationships” and “Leadership”). Scree plot suggested the structure with two factors for “Work-individual interface” (KMO = 0.57). In this domain, the “Job satisfaction” dimension consisted of only one question, while the “Work family conflicts” dimension had two questions. “Values at the workplace” consisted of four questions and the factorial solution contained a factor (KMO = 0.79). Scree plot also suggested the structure with two factors for “Health and wellness” (KMO = 0.77). For this domain, the “General health” dimension comprised one question and the “Burnout and stress” dimension consisted of four questions. For the “Offensive behaviours” domain, factor analysis obtained one factor.

**Table 2:** Results of the exploratory factor analysis for each COPSOQ II-Br domain (n = 211).

Domains	Questions	Factor 1	Factor 2
**Demands at work**			
	1A) Do you delay the delivery of your work?	**0.44**	
	1B) Do you have enough time to carry out your work tasks?	**0.64**	
	2A) Is it necessary to maintain a fast pace at work?	**0.75**	
	2B) Do you work at a high pace throughout the journey?	**0.73**	
	3A) Does your work put you in emotionally draining situations?	**0.72**	
	3B) Do you have to deal with other people’s personal problems as part of your work?	**0.72**	
**Eigenvalues**		2.73	
**% of variance**		45.6	
**Work organization and content**		**Influence and development**	**Meaning and commitment**
	4A) Do you have a high degree of influence in decisions about your work?	**0.81**	-0.02
	4B) Can you influence the amount of work assigned to you?	**0.74**	0.02
	5A) Do you have the possibility to learn new things through your work?	**0.62**	0.29
	5B) Does your work require you to take initiative?	**0.69**	0.13
	6A) Is your work meaningful?	0.11	**0.76**
	6B) Do you feel that the work you do is important?	0.02	**0.79**
	7A) Do you feel that your place of work is very important to you?	0.05	**0.75**
	7B) Would you recommend a friend to apply for a position in your workplace?	0.20	**0.72**
**Eigenvalues**		2.67	2.81
**% of variance**		20.9	35.2
**Interpersonal relationships**		**Interpersonal relationships**	**Leadership**
	8A) At your workplace, are you informed in advance about important decisions, changes, or plans for the future?	**0.63**	0.45
	8B) Do you receive all the information you need to do your job well?	**0.56**	0.52
	9A) Is your work recognized and appreciated by management?	**0.66**	0.48
	9B) Are you treated fairly at your workplace?	**0.61**	0.52
	10A) Does your work have clear objectives/goals?	**0.79**	0.20
	10B) Do you know exactly what is expected of you at work?	**0.76**	-0.03
	11A) Would you say that your immediate superior gives high priority to job satisfaction?	0.27	**0.81**
	11B) Would you say your superior is good at work planning?	0.26	**0.77**
	12A) How often is your immediate superior willing to listen to your problems at work?	0.15	**0.82**
	12B) How often do you receive help and support from your immediate superior?	0.15	**0.81**
**Eigenvalues**		1.21	5.26
**% of variance**		12.1	52.6
**Work-individual interface**		**Job satisfaction**	**Work family conflicts**
	13) What is your level of satisfaction with your work as a whole, considering all aspects?	**0.98**	-0.18
	14A) Do you feel that your work takes so much of your energy that it has a negative effect on your private life?	-0.25	**0.90**
	14B) Do you feel that your work takes up so much time that it has a negative effect on your private life?	-0.11	**0.94**
**Eigenvalues**		0.77	1.99
**% of variance**		25.8	66.4
**Values at the workplace**			
	15A) Can you trust the information that comes from your superiors?	**0.86**	
	15B) Do your superior trust that the employees will do their work well?	**0.83**	
	16A) Are conflicts resolved fairly?	**0.84**	
	16B) Is your work distributed fairly?	**0.81**	
**Eigenvalues**		2.78	
**% of variance**		69.5	
**Health and wellness**		**General health**	**Burnout and stress**
	17) In general, would you say your health is:	**0.67**	-0.61
	18A) How often have you felt physically exhausted?	-0.24	**0.82**
	18B) How often have you felt emotionally drained?	-0.02	**0.86**
	19A) How often have you felt stressed?	0.33	**0.86**
	19B) How often have you felt irritated?	0.43	**0.79**
**Eigenvalues**		0.80	3.14
**% of variance**		15.9	62.8
**Offensive behaviours**			
	20) Have you been exposed to undesired sexual attention at your workplace in the past 12 months?	**0.44**	
	21) Have you been exposed to threats of violence at your workplace in the past 12 months?	**0.84**	
	22) Have you been exposed to physical violence at your workplace in the past 12 months?	**0.68**	
	23) Have you been exposed to bullying at your workplace in the past 12 months?	**0.59**	
**Eigenvalues**		1.70	
**% of variance**		42.7	

#### Internal consistency

For ten of the 11 dimensions of COPSOQ II-Br, the Cronbach’s alpha values varied between 0.70 and 0.87; for one dimension, the Cronbach’s alpha value was less than 0.70 (“Offensive behaviours”, Cronbach’s α = 0.54) (Table 3).

**Table 3:** Measures of central tendency and variability for each COPSOQ II-Br dimension, proportion of responses in the extreme categories (floor and ceiling effect), and internal consistency (Cronbach’s α).

Domains and Dimensions (n = 211)	Mean (SD)	Median (IQR)	Range	Floor effect (%)	Ceiling effect (%)	N of items (Cronbach’s α)
**Demands at work**						
Demands at work	11.4 (4.3)	12.0 (6.0)	22.0	1.4	0.5	6 (0.76)
**Work organization and content**						
Influence and development	10.0 (3.2)	11.0 (5.0)	16.0	0.5	1.9	4 (0.70)
Meaning and commitment	13.2 (2.9)	14.0 (4.0)	16.0	0.5	27.5	4 (0.75)
**Interpersonal relationships**						
Interpersonal relationships	15.7 (5.3)	17.0 (8.0)	24.0	0.9	4.3	6 (0.86)
Leadership	10.5 (4.1)	12.0 (7.0)	16.0	1.9	10.9	4 (0.87)
**Work-individual interface**						
Job satisfaction	2.1 (0.7)	2.0 (0.0)	3.0	2.4	22.3	1 (-)
Work family conflicts	2.5 (2.0)	2.0 (4.0)	6.0	26.1	8.5	2 (0.86)
**Values at the workplace**						
Values at the workplace	10.4 (3.8)	11.0 (5.0)	16.0	1.9	7.1	4 (0.85)
**Health and wellness**						
General health	2.4 (1.0)	3.0 (1.0)	4.0	1.9	13.3	1 (-)
Burnout and stress	8.4 (3.4)	8.0 (5.0)	16.0	0.9	2.8	4 (0.87)
**Offensive behaviours**	0.5 (0.8)	0.0 (1.0)	4.0	69.2	0.5	4 (0.54)

SD: standard deviation; IQR: interquartile range.

#### Floor and Ceiling Effect

Most dimensions of the COPSOQ II-Br short version presented acceptable floor and ceiling effects, except for the floor effect in the “Work family conflicts” dimension (26.1%) and, for the ceiling effect in the dimensions “Meaning and commitment” (27.5%) and “Job satisfaction” (22.3%) (Table 3).

##### Test-retest reliability and measurement error

All 11 dimensions showed acceptable reliability, with ICC_(3,1)_ between 0.71 and 0.81. The SEM ranged from 0.6 to 2.2, being lower for the dimensions “Offensive behaviors” and “Work family conflicts”, and higher for the dimensions “Demands at work”, “Interpersonal relationships” and “Influence and development”.

Minimal detectable change (MDC) ranged from 0.8 to 5.9 indicating, for example, that a change in the “Interpersonal relationships” dimension score must be at least 6 points to represent a real change for the individual (Table 4).

**Table 4:** Mean values and standard deviation (SD) in the test and retest (n = 157), intraclass correlation coefficient (ICC), standard error of measurement (SEM), and minimum detectable change (MDC) of the COPSOQ II-Br short version.

Domains and Dimensions (n = 157)	Mean (SD) Test	Mean (SD) Retest	ICC_(3,1)_ (95% CI)	SEM	MDC
**Demands at work**					
Demands at work	11.1 (4.4)	11.3 (3.9)	0.81 (0.75–0.86)	2.2	4.5
**Work organization and content**					
Influence and development	9.8 (3.3)	9.8 (3.2)	0.74 (0.66–0.80)	1.8	3.9
Meaning and commitment	13.2 (3.0)	13.2 (3.0)	0.76 (0.69–0.82)	1.5	3.4
**Interpersonal relationships**					
Interpersonal relationships	16.1 (5.4)	15.8 (5.2)	0.78 (0.70–0.83)	2.0	5.9
Leadership	11.0 (3.9)	10.7 (3.8)	0.76 (0.69–0.82)	1.4	4.4
**Work-individual interface**					
Job satisfaction	2.1 (0.7)	2.1 (0.7)	0.71 (0.62–0.78)	-	0.9
Work family conflicts	2.4 (2.0)	2.6 (1.9)	0.79 (0.72–0.84)	0.8	2.2
**Values at the workplace**					
Values at the workplace	10.6 (4.1)	10.8 (3.6)	0.71 (0.63–0.78)	1.6	5.1
**Health and wellness**					
General health	2.4 (1.0)	2.5 (0.9)	0.73 (0.64–0.79)	-	1.2
Burnout and stress	8.3 (3.5)	8.4 (3.5)	0.79 (0.73–0.85)	1.3	3.7
**Offensive behaviours**	0.4 (0.8)	0.4 (0.8)	0.80 (0.74–0.85)	0.6	0.8

- not applicable; SD: standard deviation; CI: confidence interval.

##### Construct validity

The analysis revealed significant and positive correlations between the dimensions of the COPSOQ II-Br and the JCQ scales. The point-biserial correlation coefficient between the presence of musculoskeletal symptoms and the dimensions of the COPSOQ II-Br were significantly and positively correlated for three dimensions (“Demands at work”, “Work family conflits” and “Burnout and stress”). We also found a significant and negative correlation between the presence of musculoskeletal symptoms and the dimensions “Interpersonal relationships”, “Job satisfaction” and “General health” of COPSOQ II-Br. These results confimed all the hypotheses formulated (Table 5).

**Table 5:** Spearman’s correlation coefficient (rs) between the Job Content Questionnaire (JCQ) scales, Point-biserial correlation coefficient (rpb) between musculoskeletal symptoms in the past 12 months and 7 days, and Spearman’s correlation coefficient (rs) between the number of regions with musculoskeletal symptoms in the past 12 months and 7 days and the dimensions of the COPSOQ II-Br short version.

Dimensions COPSOQ II	Job Content Questionnaire			Musculoskeletal symptoms					
	
	Work demands	Control	Social support	Neck		Low back		Number of regions with symptoms	
		
				r_pb_ 12 months	r_pb_ 7 days	r_pb_ 12 months	r_pb_ 7 days	r_s_ 12 months	r_s_ 7 days
**Demands at work**									
Demands at work	0.63^a^	0.23^a^	-0.37^a^	0.29^a^	0.25^a^	0.24^a^	0.20^a^	0.37^a^	0.28^a^
**Work organization and content**									
Influence and development	0.20^a^	0.66^a^	-0.07	0.17^a^	0.11	0.15^a^	0.08	0.11	0.07^a^
Meaning and commitment	-0.17^a^	0.28^a^	0.30^a^	-0.08	-0.04	-0.06	-0.03	-0.17^a^	-0.15^a^
**Interpersonal relationships**									
Interpersonal relationships	-0.33^a^	0.25^a^	0.36^a^	-0.02	-0.03	-0.08	-0.11	-0.15^a^	-0.17^a^
Leadership	-0.28^a^	0.12	0.43^a^	-0.04	0.01	0.01	-0.04	-0.14	-0.15^a^
**Work-individual interface**									
Job satisfaction	-0.38^a^	0.19^a^	0.39^a^	-0.12	-0.06	-0.14^a^	-0.08	-0.22^a^	-0.18^a^
Work family conflicts	0.42^a^	-0.05	-0.38^a^	0.24^a^	0.20^a^	0.18^a^	0.16^a^	0.36^a^	0.32^a^
**Values at the workplace**									
Values at the workplace	-0.36^a^	0.19^a^	0.46^a^	-0.07	-0.03	0.01	-0.06	-0.21^a^	-0.21^a^
**Health and wellness**									
General health	-0.21^a^	0.20^a^	0.16	-0.17^a^	-0.07	-0.16^a^	-0.15^a^	-0.35^a^	-0.29^a^
Burnout and stress	0.41^a^	-0.01	-0.43^a^	0.29^a^	0.20^a^	0.21^a^	0.20^a^	0.31^a^	0.26^a^
**Offensive behaviours**	0.22^a^	-0.06	-0.20^a^	0.05	0.05	0.17^a^	0.02	0.08	0.01

^a^ p < 0.05.

## DISCUSSION

The present study cross-culturally adapted the short version of the COPSOQ II into Brazilian Portuguese and analyzed its psychometric properties.

The exploratory factor analysis revealed a questionnaire structure composed of seven domains and 11 dimensions (40 questions). The Brazilian study on the medium version of COPSOQ I obtained a structural model with four domains, 17 dimensions and 53 questions^18^. The study by Lima et al^19^. about the Spanish medium version of COPSOQ-ISTAS21 II (six domains, 21 dimensions and 70 questions) resulted in 13 dimensions and 70 questions^19^. Our study, therefore, presents an acceptable structural validity result, respecting the original theoretical model of COPSOQ^16^.

Regarding internal consistency, the results showed that the Cronbach’s alpha values, in general, reached those recommended in the literature (above 0.70), excepting the “Offensive behaviours” dimension. These findings are in agreement with the results of other studies^8^.

As for the floor and ceiling effect, our results for the floor effect are similar to those found in the original Danish study^16^ and in the study that cross-culturally adaptated the instrument to Portuguese in Portugal^14^, both evaluating the long version of COPSOQ. Both studies found acceptable floor effects (less than 15%) for all dimensions, except for “Work family conflicts”. Our values for the ceiling effect, in turn, differ from studies in the literature^14,16^, as two dimensions presented this effect. This finding can be explained by the type of worker included in the sample, since teachers, managers and administrative positions tend to score higher for these dimensions^15^.

The test-retest reliability measurements showed that all dimensions had good consistency (0.71 to 0.81), result similar to those found by Aminian et al.^13^ (0.75 to 0.89) and Rosário et al.^14^ (0.71 to 0.93).

Studies that tested the psychometric properties of COPSOQ show good results in terms of internal consistency^8^and test-retest reliability^13,14^, but most of them evaluated the first version of the questionnaire (COPSOQ I)^9^, in its medium^8,10,12,18,19^and long^9,14^ versions, making adaptations in the instrument^8^.

The present study assessed construct validity by the correlations between the dimensions of the COPSOQ II-Br and the JCQ scales. These findings are in accordance with the COPSOQ proposal, which addresses, among others, the Karasek theory with equivalent questions in the questionnaire^15,16^.

The association between COPSOQ II-Br and musculoskeletal symptoms presetend adequate results, in agreement with other studies in the literature that noted the association between these psychosocial aspects and musculoskeletal symptoms^27^. Interestingly, in our study, some of the dimensions showed a negative correlation with the presence of musculoskeletal symptoms, i. e., the greater the presence of these psychosocial aspects in the workplace the lower the proportion of symptoms.

Hauke et al.^27^ found that psychosocial working conditions such as low social support, job control, decision-making power, job satisfaction and high job demands, were associated with increased risk of lower back and neck/shoulder pain. A cross-sectional study conducted in Brazil, in turn, showed that low social support, high psychological demand and low job control increased the prevalence of multiregional symptoms^29^. Thus, the results obtained in this study were consistent with the literature findings.

Some limitations of this study concern aspects related to data collection. First, the online questionnaire had a low response rate (15%) when compared to the in-person format (75%). Second, the sample lacked balance regarding sex, which may have influenced the responses. Finally, the study did not include industry and retail workers, sectors that should be investigated in future studies.

Therefore, this study was able to culturally-adapt the short version of COPSOQ II into Brazilian Portuguese and test its psychometric measures, whose results were considered suitable for using the instrument to assess the health of the population of Brazilian workers.

## Footnotes

a Gonçalves JS. Os aspectos psicossociais no trabalho [tese]. São Carlos: Universidade Federal de São Carlos; 2019 [cited 2021 Jun 19]. Available from: https://repositorio.ufscar.br/handle/ufscar/11763
